# The superior cervical ganglion is involved in chronic chemoreflex sensitization during recovery from acute lung injury

**DOI:** 10.3389/fphys.2023.1101408

**Published:** 2023-02-08

**Authors:** Kajal Kamra, Nikolay Karpuk, Irving H. Zucker, Harold D. Schultz, Han-Jun Wang

**Affiliations:** ^1^ Department of Cellular and Integrative Physiology, University of Nebraska Medical Center, Omaha, NE, United States; ^2^ Department of Anesthesiology, University of Nebraska Medical Center, Omaha, NE, United States

**Keywords:** acute respiratory distress syndrome, bleomycin, chemoreceptors, carotid body, glomus cells

## Abstract

**Introduction:** Acute lung injury (ALI) initiates an inflammatory cascade that impairs gas exchange, induces hypoxemia, and causes an increase in respiratory rate (f_R_). This stimulates the carotid body (CB) chemoreflex, a fundamental protective reflex that maintains oxygen homeostasis. Our previous study indicated that the chemoreflex is sensitized during the recovery from ALI. The superior cervical ganglion (SCG) is known to innervate the CB, and its electrical stimulation has been shown to significantly sensitize the chemoreflex in hypertensive and normotensive rats. We hypothesized that the SCG is involved in the chemoreflex sensitization post-ALI.

**Methods:** We performed a bilateral SCG ganglionectomy (SCGx) or sham-SCGx (Sx) in male Sprague Dawley rats 2 weeks before inducing ALI (Week −2 i.e., W-2). ALI was induced using a single intra-tracheal instillation of bleomycin (bleo) (day 1). Resting-f_R_, V_t_ (Tidal Volume), and V̇ _E_ (Minute Ventilation) were measured. The chemoreflex response to hypoxia (10% O_2_, 0% CO_2_) and normoxic-hypercapnia (21% O_2_, 5% CO_2_) were measured before surgery on W (−3), before bleo administration on W0 and on W4 post-bleo using whole-body plethysmography (WBP).

**Results:** SCGx did not affect resting f_R_, V_t_ and V̇_E_ as well as the chemoreflex responses to hypoxia and normoxic hypercapnia in either group prior to bleo. There was no significant difference in ALI-induced increase in resting f_R_ between Sx and SCGx rats at W1 post-bleo. At W4 post-bleo, there were no significant differences in resting f_R_, V_t_, and V̇_E_ between Sx and SCGx rats. Consistent with our previous study, we observed a sensitized chemoreflex (delta f_R_) in response to hypoxia and normoxic hypercapnia in Sx rats at W4 post-bleo. However, at the same time, compared to Sx rats, the chemoreflex sensitivity was significantly less in SCGx rats in response to either hypoxia or normoxic hypercapnia.

**Discussion:** These data suggest that SCG is involved in the chemoreflex sensitization during ALI recovery. Further understanding of the underlying mechanism will provide important information for the long-term goal of developing novel targeted therapeutic approaches to pulmonary diseases to improve clinical outcomes.

## Introduction

Acute lung injury (ALI) and its clinical correlate, the acute respiratory distress syndrome (ARDS) affects approximately 200,000 new cases each year in the US alone and accounts for 10% of Intensive Care Unit admissions with a high mortality and morbidity (Johnson and Matthay, 2010; Conde et al., 2014). ARDS results in disruption of the normal alveolar-capillary endothelial barrier leading to pulmonary interstitial and alveolar edema thus impairing gas exchange (Ghio et al., 2001; Young et al., 2019; Spinelli et al., 2020). Hypoxemia resulting from ALI stimulates carotid bodies (CBs) to induce a reflexive increase in respiratory rate (fR) (i.e., chemoreflex), which is a fundamental protective mechanism that maintains oxygen homeostasis (Bernard et al., 1994; Jacono et al., 2006; Spinelli et al., 2020). Our laboratory has previously demonstrated that while f_R_ recovers, the chemoreflex remains chronically sensitized during the recovery from ALI (Kamra et al., 2022). The chemoreflexes are important modulators of sympathetic activation. It is well established that acute and/or chronic activation of the CBs enhance sympathetic drive. Excessive sympathetic outflow can lead to cardiac arrhythmias, cardio-renal syndrome, metabolic syndrome, Type 2 diabetes, and deterioration of cardiac function (Conde et al., 2014; Del Rio et al., 2017; Cunha-Guimaraes et al., 2020). The neural mechanism that drives the chronic chemoreflex sensitization during the recovery from the ALI is not fully understood.

The postganglionic sympathetic fibers of the superior cervical ganglion (SCG) innervate a series of inter-related structures including the internal carotid artery, CB, and carotid sinus (Wang et al., 2018). Previously, it has been demonstrated that electrical stimulation of the sympathetic efferent nerves that originate in the SCG can sensitize the CB chemoreflex in hypertensive and normotensive rats (Felippe et al., 2022). Moreover, superior cervical ganglionectomy (SCGx) has been shown to attenuate the sensitivity of the chemoreflex in the spontaneously hypertensive rat suggesting that the CB hyper-excitability may be driven by excessive activity of its sympathetic innervation (Felippe et al., 2022). The specific objective of our study was to investigate the role of the SCG in modulating chronic chemoreflex sensitization during recovery from ALI.

## Methods

**FIGURE 1:**
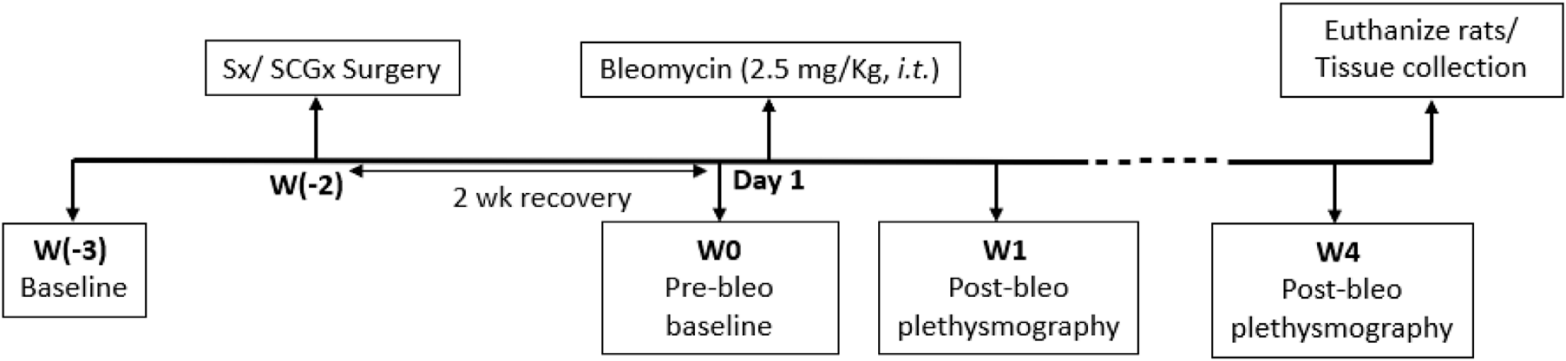
Timeline showing experimental design.

All experimental protocols were approved by the Institutional Animal Care and Use Committee (IACUC) of the University of Nebraska Medical Center (protocol ID no. 17-006-03 FC). Eighteen adult male Sprague-Dawley rats (250–350 g) were used for these experiments. Animals were housed on-site and given a 1-week acclimation period prior to experimentation. Food and water were supplied *ad libitum*, and rats were kept on 12-h light/dark cycles. All animal experimentation (collection of ventilatory parameters during rest and during hypoxic/normoxic-hypercapnic gas exposure) was performed during the day (9:00-16:00 h). Delivery of bleomycin (bleo) was performed within our animal housing center. At the end of the experimental protocol, all animals were humanely euthanized with an overdose of pentobarbital sodium (150 mg/kg, IV). Euthanasia was confirmed by removal of vital organs and lung tissue was collected for further analysis. An experimental timeline is shown in Figure 1.

### Drugs and chemicals

Bleomycin sulphate (or, bleomycin, bleo) was purchased from Enzo Life Sciences (New York, United States). Bleo was dissolved in saline for intra-tracheal administration. This procedure was performed within the animal housing center.

### Rat model of lung injury

Rats were randomized into two experimental groups and evaluated at four time points-baseline (W-3), SCG ablation (SCGx) or sham surgery (Sx) (W-2), recovery and pre-bleomycin instillation for both groups (W0), 1-week post-instillation both groups (W1) and 4 weeks post-instillation (W4): Sx rats (n = 10) and SCGx rats (n = 8). Bleo (2.5 mg/kg, ∼0.15 mL) was instilled on day 1 of W0 intra-tracheally under 2%–3% isoflurane anesthesia after evaluation.

### Superior cervical ganglionectomy

Rats were anesthetized, intubated, and ventilated with isoflurane. Using sterile techniques, a 4-cm vertical incision was made along the ventral surface of the neck. Muscles were retracted to expose the common carotid artery. The carotid artery was then bluntly dissected up to the level of its bifurcation into the external and internal carotid arteries. For SCGx, the SCG was identified behind the carotid bifurcation and was gently pulled until its avulsion. For Sx, muscles were retracted, and the common carotid bifurcation was dissected to expose the SCG without touching or disturbing the SCG. The skin was closed with 4–0 nylon, using a simple interrupted suture, placing a stitch every 4–5 mm (Savastano et al., 2010). The rats were placed in a heated cage until fully awake.

### Breathing and ventilatory chemoreflex function at rest

Unrestrained whole-body plethysmography was utilized to measure ventilatory parameters-respiratory rate (f_R_), tidal volume (V_t_) and minute ventilation (V̇_E_) in conscious rats by using signals from a differential-pressure transducer (DLP 2.5, Harvard Apparatus), amplified, and connected to a data acquisition system PowerLab 35 Series managed by LabChart (v8.1.5) software (ADInstruments, Colorado, United States). Rats were acclimated to the plethysmograph chamber for 1 h each for two consecutive days prior to recordings. Ventilatory parameters were not recorded during the acclimatization sessions. The plethysmograph chambers used for this study were custom-made (Midwest Plastics Inc., Nebraska, United States) and were 10, 10.5 and 20 cm in height, width, and length, respectively. The volume channel (i.e., flow integration) was calibrated by pushing 5 mL of air using a syringe before the start of the recording. During recordings, a constant flow of gas at 3 L/min was maintained to avoid an increase in humidity, temperature and CO_2_ levels using a manually operated flow meter (Precision Medical, Northampton, PA, United States). Body weight (in grams) of rats was recorded prior to each experiment. In the resting state, rats were exposed to normoxia (21% O_2_, 0% CO_2_) for baseline measurements followed by two different gas challenges-hypoxia (10% O_2_, 0% CO_2_) and normoxic hypercapnia (21% O_2_, 5% CO_2_) balanced by N_2_. The order of gas challenge was randomized and was maintained for 5 minutes. The last 1-min segment without any artifacts was used for analysis. A normoxic exposure of a minimum of 10 min or more was used in between challenges. All resting ventilatory parameters considered for analysis were recorded when the rats were awake and stationary (no activity-related events recorded in LabChart8 raw data file). V̇_E_ was calculated as the product of f_R_ and V_t_. V_t_ and V̇_E_ were normalized to bodyweight. A 30-min recording without artifacts was recorded for all rats while they breathed room air. This was used to measure all resting ventilatory parameters and to manually extract apneas, hypopneas, sighs, and post-sigh apneas from LabChart8 raw data files. Apneas were defined as the cessation of breathing for at least three respiratory cycles, as determined by respiratory rate for the prior 10 s; hypopneas were defined as reductions in breath amplitude 50% of the average cycle amplitude of the preceding 10 s of regular breathing; post-sigh apneas were defined as the cessation of breathing for at least three respiratory cycles immediately after a sigh (Díaz et al., 2020). Apneas and hypopneas were expressed as Apnea-Hypopnea Index (AHI, events/hour). Sighs and post-sigh apneas were also expressed as events/hour.

### Statistical analysis

Data analysis in text, tables and figures are presented as mean ± SD. Statistical evaluation was analyzed using GraphPad Prism (GraphPad Software, San Diego, CA. Version 8). Comparisons between conditions (gas challenges) and for comparisons between groups (Sx and SCGx) were done using the repeated-measures two-way ANOVA with Bonferroni corrections for multiple comparisons with *p* < 0.05 being statistically significant. Sample size represents number of animals.

## Results

### SCGx does not change the baseline resting ventilatory parameters in rats prior to bleo treatment.

**FIGURE 2:**
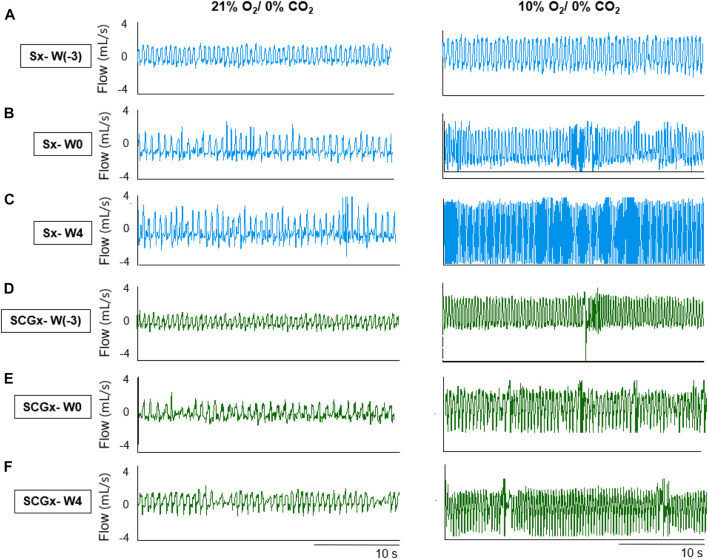
Representative recordings of resting breathing at normoxia (left) and 10% hypoxia (right) obtained in one rat per experimental group: **(A–C)** Sx (in blue) at W0, W1 and W4, respectively; **(D–F)** SCGx (in green) at W0, W1 and W4, respectively.

**FIGURE 3:**
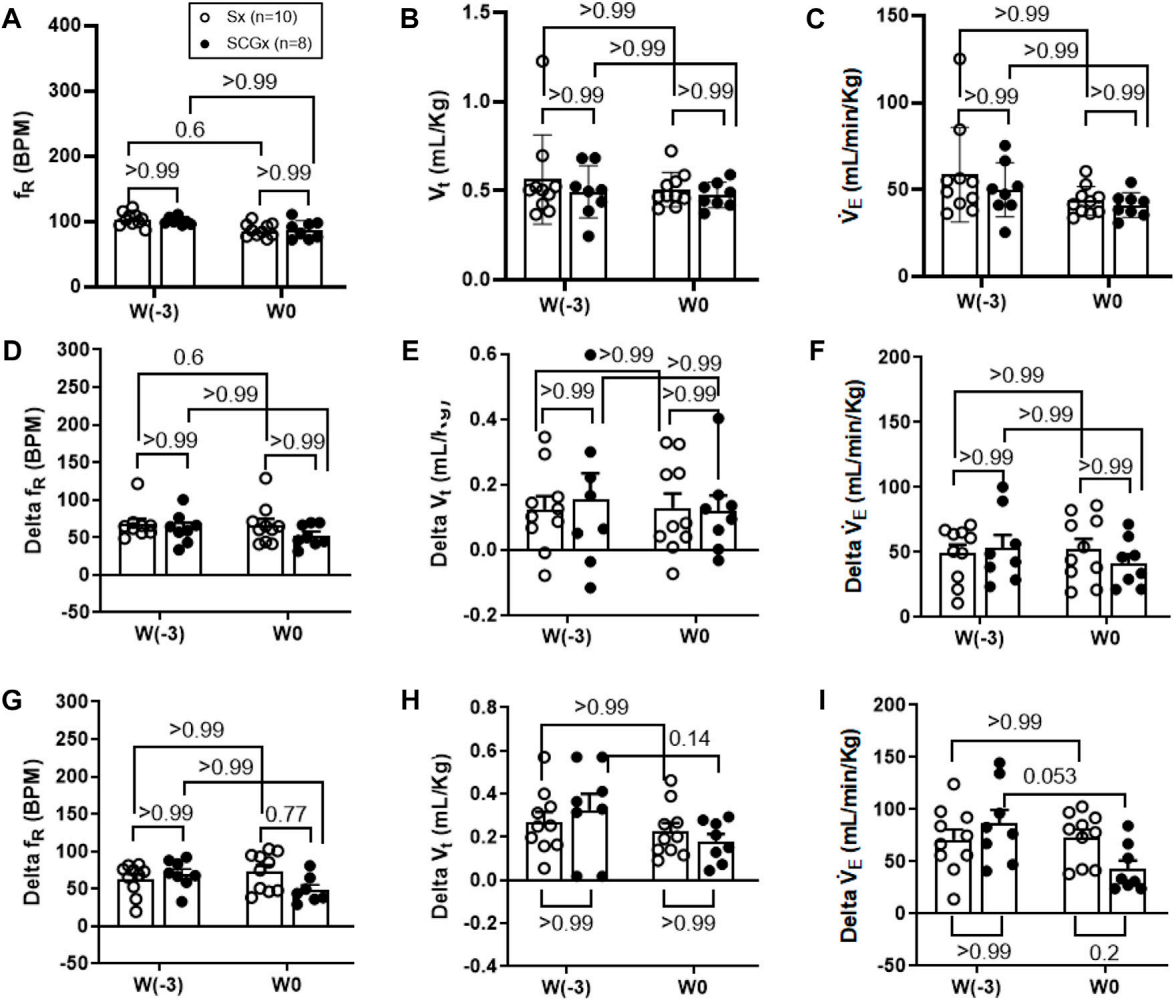
Effect of Sx (n = 10) and SCGx (n = 8) on both resting ventilatory parameters **(A–C)** and chemoreflex activation in response to either 10% hypoxia **(D–F)** or to 5% normoxic-hypercapnia **(G–I)** in rats prior to bleo-treatment. Two-way ANOVA was applied using Bonferroni multiple comparisons, Values are mean ± SD; **(A)** Respiratory rate (f_R_); **(B)** Tidal volume (V_t_); **(C)** Minute ventilation (V̇ _E_). **(D–F)** changes in ventilatory parameters including f_R,_ V_t_ and V`˙ _E_ in response to 10% hypoxia in rats prior to bleo-treatment. **(G–I)** changes in ventilatory parameters including f_R,_ V_t_ and V̇ _E_ in response to 5% normoxic-hypercapnia in rats prior to bleo-treatment.

The resting ventilatory parameters for Sx and SCGx rats were recorded during the 30-min exposure to room air-before surgery (W-3) and 2 weeks post-recovery from the Sx or SCGx surgery (W0). There were no significant changes in resting f_R_, V_t_ and V̇_E_ before and after Sx or SCGx surgery (Sx: *p* > 0.99 (W-3 vs W0) and SCGx: *p* > 0.99 (W-3 vs. W0) (Figure 2, Figure 3A).

### SCGx does not change the chemoreflex response to hypoxia in rats prior to bleo treatment.

The chemoreflex was activated by challenging the rats with 10% hypoxia for a duration of 5 minutes and ventilatory parameters were assessed. The chemoreflex was assessed by measuring the absolute difference between 21% O_2_/0% CO_2_ and 10% O_2_/0% CO_2_. At baseline before the surgery (W-3), f_R_ increased in response to 10% O_2_ in Sx and SCGx rats by 61 ± 30 bpm and 62 ± 20 bpm, respectively. After 2 weeks of surgery (W0) the chemoreflex in response to 10% hypoxia in Sx and SCGx rats increased by 66 ± 26 bpm and 52 ± 14 bpm, respectively. There was no significant difference in these chemoreflex-evoked f_R_ responses to hypoxia in either group before or after Sx/SCGx (W-3 vs W0) (Figure 3D). Similarly, when W-3 was compared to W0, Sx and SCGx exhibited no significant differences in delta V_t_ (Sx: *p* > 0.99 and SCGx: *p* > 0.99) (Figure 3E) and the delta V̇_E_ (Sx: *p* > 0.99 and SCGx: *p* > 0.99) (Figure 3F).

### SCGx does not change the chemoreflex response to normoxic-hypercapnia in rats prior to bleo treatment.

In the same groups of rats, both peripheral and central chemoreflexes were activated by challenging the rats with 5% CO_2_/21% O_2_ for a duration of 5 minutes. The chemoreflex was assessed by measuring the absolute difference between 21% O_2_/0% CO_2_ and 21% O_2_/5% CO_2_. At baseline before the surgery (W-3), f_R_ increased in response to 21% O_2_/5% CO_2_ in Sx and SCGx rats by 63 ± 21 bpm and 70 ± 19 bpm, respectively. After 2 weeks of recovery, the chemoreflex in response to 5% normoxic-hypercapnia in Sx and SCGx rats increased by 74 ± 25 bpm and 49 ± 18 bpm, respectively. There were no significant differences in these chemoreflex-evoked f_R_ responses to normoxic-hypercapnia in either group before and after Sx/SCGx (W-3 vs. W0) (Figure 3G). Similarly, when W-3 was compared to W0, Sx/SCGx showed no significant differences in delta V_t_ (Sx: *p* > 0.99 and SCGx: *p* > 0.99) (Figure 3H) and delta V̇_E_ (Sx: *p* > 0.99 and SCGx: *p* > 0.99) (Figure 3I).

### SCGx did not change baseline resting ventilatory parameters in rats at W1 post-bleo (ALI).

**FIGURE 4:**
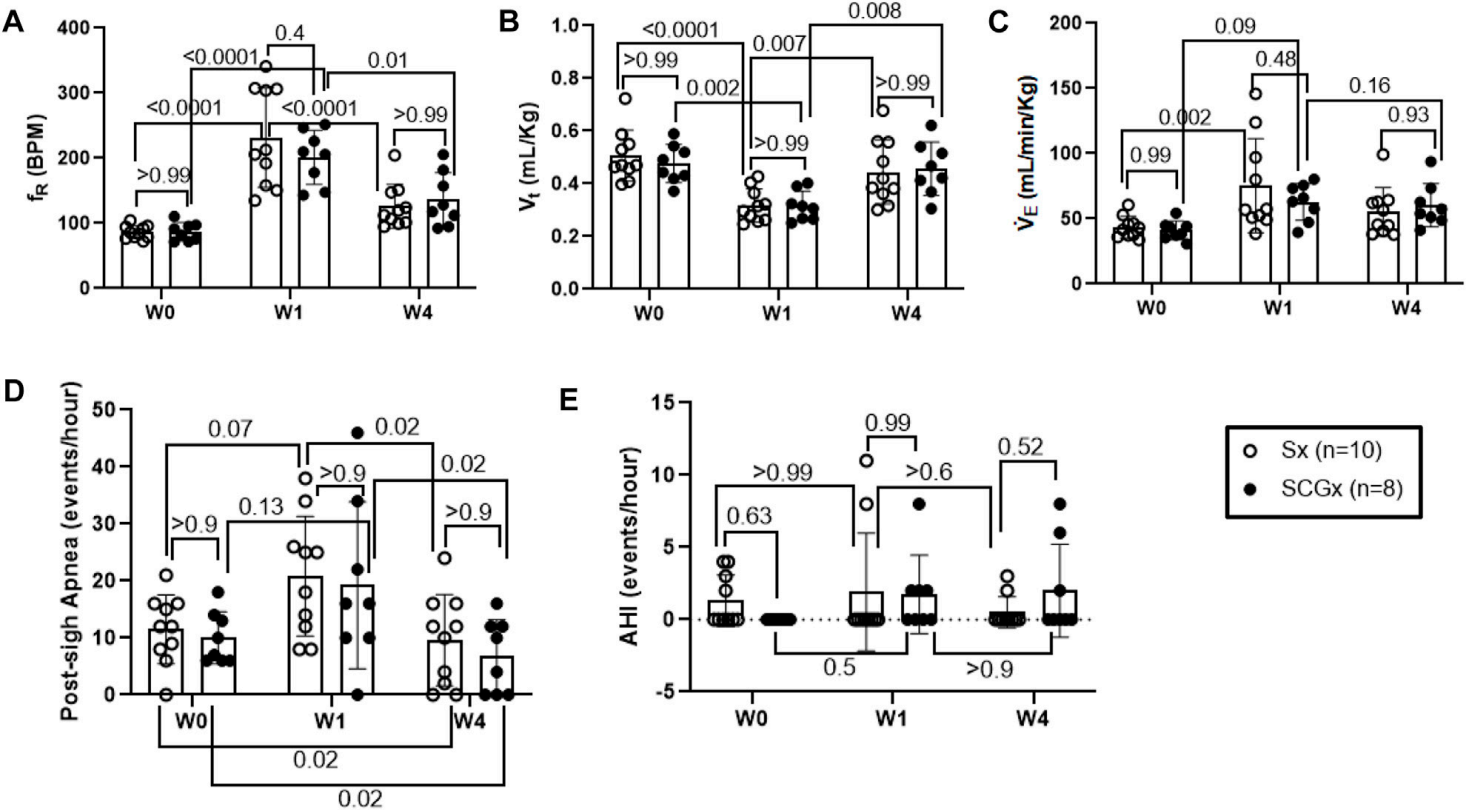
Effect of Sx (n = 10) and SCGx (n = 8) on resting ventilatory parameters-**(A)** Respiratory rate (f_R_); **(B)** Tidal volume (V_t_); **(C)** Minute ventilation (V̇ _E_) and, **(D)** Post-sigh Apneas and **(E)** Apnea-hypopnea Index (AHI) in bleo rats at W1 and W4 post-bleo. Two-way ANOVA was applied using Bonferroni multiple comparisons, Values are mean ± SD.

At day 1 of the experimental timeline, we intra-tracheally instilled bleo to both groups of rats and measured resting ventilatory parameters at W1-post-bleo instillation. As noted in Figure 4A, in response to bleo instillation, the resting f_R_ increased (*p* < 0.0001) in both Sx- (231 ± 76 bpm) and SCGx rats (201 ± 42 bpm) at W1 post-bleo administration as compared to their pre-bleo baseline at W0 (Sx: 86 ± 10 bpm; and SCGx: 87 ± 14 bpm). There was no difference between the Sx and SCGx groups in resting f_R_ before or after bleo (Sx vs. SCGx: *p* > 0.99 (W0) and *p* = 0.37 (W1)). Similarly, there were no differences in resting V_t_ and V̇_E_ between the two groups before or after bleo [V_t_: (Sx vs SCGx: *p* > 0.99 (W0) and *p* > 0.99 (W1)); V̇_E_ (Sx vs SCGx: *p* > 0.99 (W0) and *p* = 0.84 (W1))] (Figures 4B,C).

### SCGx did not change baseline resting ventilatory parameters in rats at W4 post-bleo.

At W4 post-bleo, the resting f_R_ was largely restored for both Sx group (126 ± 34 bpm) and SCGx group (136 ± 41 bpm) compared to W0 (Sx: 86 ± 10 bpm; and SCGx: 87 ± 14 bpm). There was no significant change in the resting ventilatory parameters between Sx and SCGx groups at W4 post-bleo (f_R_- Sx vs SCGx: *p* > 0.99, Figure 4A; V_t_- Sx vs SCGx: *p* > 0.99; Figure 4B; V̇_E_ - Sx vs SCGx: *p* = 0.84; Figure 4C).

### SCGx did not change the occurrence of apneic events in rats at W1 and W4 post-bleo.

The 30 minute-raw data recorded during normoxic gas exposure were utilized to manually extract apneas from Sx and SCGx rats at W0, W1 and W4 post-bleo. The occurrence of apneas and post-sigh apneas tended to increase but did not reach statistical significance in Sx and SCGx rats at W1 post-bleo as compared to W0. However, it significantly decreased (*p* > 0.02) for both Sx and SCGx rats at W4 post-bleo compared to W0 (Figure 4D). There were no significant changes in AHI for either Sx or SCGx groups at W1 or W4 post-bleo compared to W0 (Figure 4E).

### SCGx attenuated chemoreflex sensitization to both hypoxia and normoxic-hypercapnia W4 post-bleo

**FIGURE 5:**
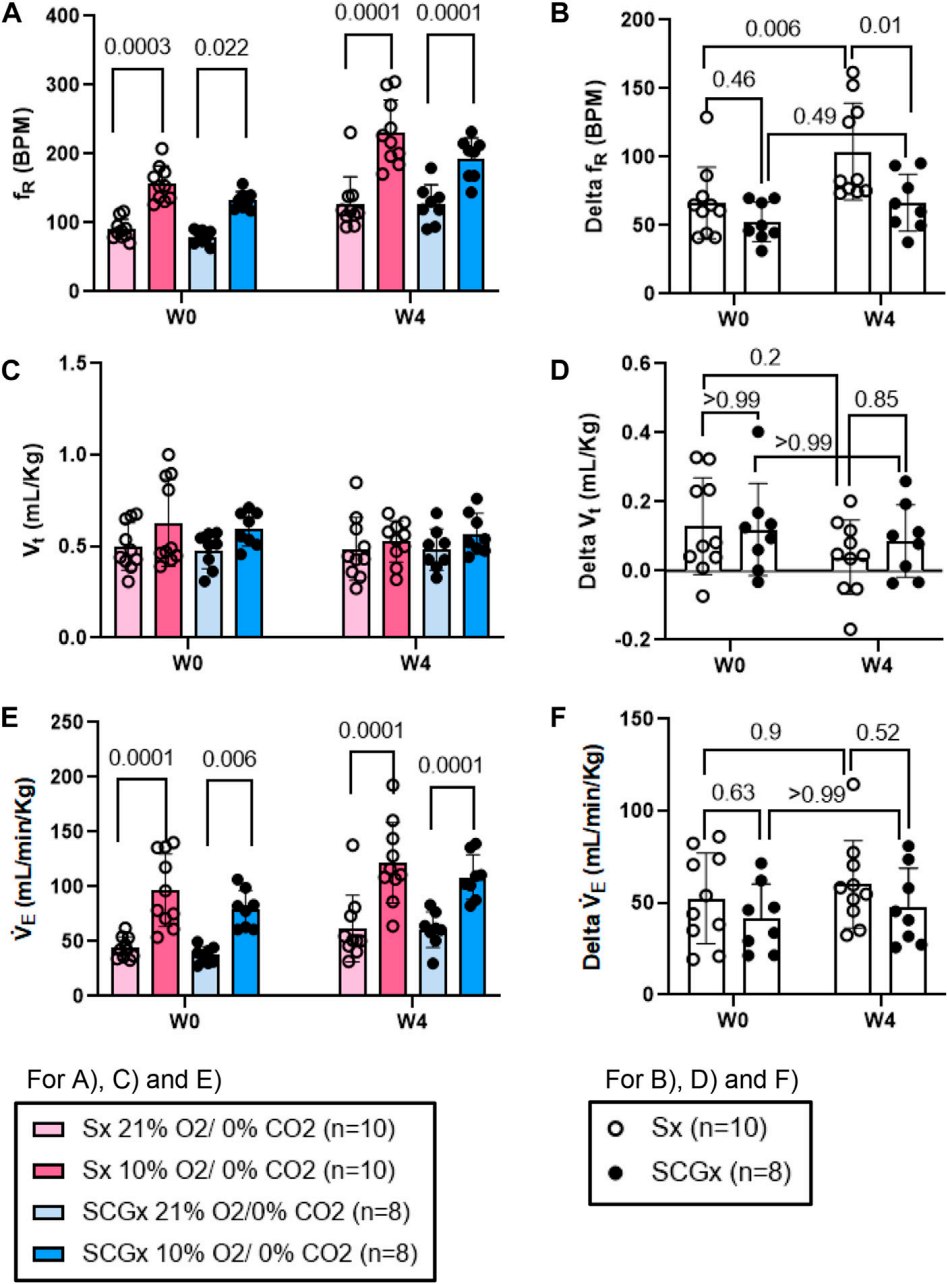
Effect of SCGx (n = 8) on sensitized chemoreflex activity on ventilatory parameters in response to hypoxia at W4 post-bleo. Two-way ANOVA was applied using Bonferroni multiple comparisons, Values are mean ± SD; **(A)** Respiratory rate (f_R_); **(B)** Delta f_R_; **(C)** Tidal volume (V_t_); **(D)** Delta V_t_; **(E)** Minute ventilation (V̇ _E_); **(F)** Delta V̇ _E_.

The chemoreflex responses to 10% hypoxia and 5% normoxic-hypercapnia were assessed by measuring the absolute difference between 21% O_2_/0% CO_2_ and 10% O_2_/0% CO_2_ or, 21% O_2_/5% CO_2_-induced responses. At W0 both Sx and SCGx rats exhibited similar changes in f_R_, V_t_ and V̇_E_ in response to 10% hypoxia (Figures 5A, C, E). Compared to W0, the changes in f_R_ and V̇_E_ in response to 10% hypoxia at W4 post-bleo were significantly elevated in Sx rats (Figures 5B, F). However, the change in f_R_ in response to 10% hypoxia at W4 post-bleo was significantly attenuated in SCGx rats compared to Sx rats (Figure 5B). The changes in V_t_ and V̇_E_ in response to 10% hypoxia were not statistically significant between Sx and SCGx groups at W4 post-bleo (Figures 5D, F).

**FIGURE 6:**
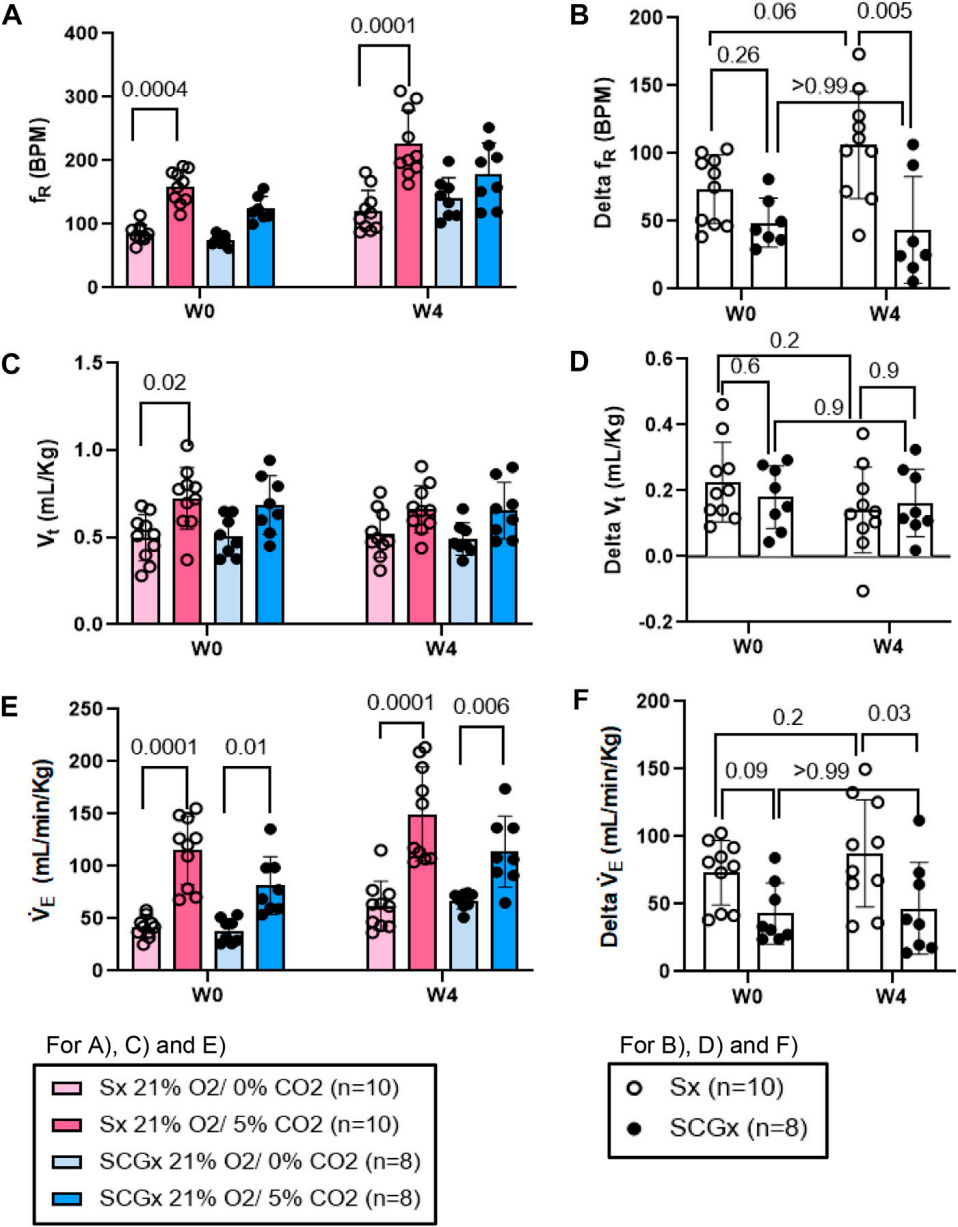
Effect of SCGx (n = 8) on sensitized chemoreflex activity on ventilatory parameters in response to normoxic-hypercapnia at W4 post-bleo. Two-way ANOVA was applied using Bonferroni multiple comparison, Values are mean ± SD; **(A)** Respiratory rate (f_R_); **(B)** Delta f_R_; **(C)** Tidal volume (V_t_); **(D)** Delta V_t_; **(E)** Minute ventilation (V̇_E_); **(F)** Delta V̇ _E_.

Similarly, compared to W0, the change in f_R_ in response to normoxic-hypercapnic gas challenge at W4 post-bleo was significantly elevated in Sx rats. The change in f_R_ in response to normoxic-hypercapnic gas challenge at W4 post-bleo was significantly attenuated in SCGx rats compared to Sx rats (Figures 6A, B). Compared to Sx rats, we also observed attenuated delta V̇_E_ (Figures 6E, F) in response to normoxic-hypercapnic gas challenge in SCGx rats at W4 post-bleo due to a blunted delta f_R_ with no significant change in delta V_t_ (Figures 6C, D).

## Discussion

In this study, we examined the role of the SCG in mediating chronic sensitization during recovery from the ALI. The major findings of this study are as follows: 1) SCGx does not change the baseline resting ventilatory parameters in rats prior to bleo treatment; 2) SCGx does not change the chemoreflex response to 10% hypoxia and 5% normoxic-hypercapnia in rats prior to bleo treatment; 3) SCGx does not change the baseline ventilatory parameters in bleo-rats at W1 post-bleo; 4) SCGx does not change the baseline resting ventilatory parameters in bleo-rats during the recovery from ALI at W4 post-bleo; 5) SCGx does not change the occurrence of apneic events in bleo-rats at W1 and W4 post-bleo; 6) SCGx attenuates chemoreflex sensitization during the recovery from ALI at W4 post-bleo.

ALI/ARDS occurs as a result of a cascade of inflammatory cellular events due to a compromised alveloar-epithelial membrane in the lung (Tsushima et al., 2009). It remains one of the leading causes of increased morbidity in the United States (Goss et al., 2003). This results in improper gas exchange leading to increased breathing frequency and eventually, hypoxemia, thus, activating a chemoreflex response during ALI (Bernard et al., 1994; Jacono et al., 2006; Spinelli et al., 2020). Furthermore, we have previously shown that there were enhanced fR responses to both hypoxia and normoxia-hypercapnia at W4 post-bleo even after the resting f_R_ is restored to the normal range (Kamra et al., 2022). Our current data in Figures 5, 6 further recapitulated this phenomenon. These data indicated that the chemoreflex remains sensitized during the recovery from ALI. It should be noted that the response of the carotid bodies to hypoxia during ALI is a hyperbolic function of arterial PO_2_, meaning that the magnitude of the response to a given decrease in arterial PaO_2_ depends greatly on the baseline (pre-stimulus) PaO_2_. Animals with ALI are hypoxic at baseline; an increase in hypoxic ventilatory reflex could be theoretically explained by the fact that their resting PaO_2_ is lower than that of healthy rats. However, our previously published data (Kitzerow et al., 2022) reported that resting PaO_2_ level was restored to nearly normal value even 2 weeks after intra-tracheal instillation of the same dose of bleomycin (i.e., 2.5 mg/kg). Therefore, the enhanced chemoreflex at W4 post-bleo is less likely due to any PaO_2_ factor. That said, the neural mechanisms underlying the chemoreflex sensitization during the recovery from ALI remain unknown.

SCG is located at the cervical sympathetic chain, which contains post-ganglionic sympathetic fibers (Tang et al., 1995a; Tang et al., 1995b; Tang et al., 1995c; Llewellyn-Smith et al., 1998). Multiple studies provide evidence that post-ganglionic fibrers from the SCG innervate a series of inter-related structures including the carotid body and carotid sinus, tongue and upper airway (Flett and Bell, 1991; O’Halloran et al., 1996; Wang et al., 2018), and brainstem nuclei, such as the NTS (Gallardo et al., 1984; Hughes-Davis et al., 2005). Felippe et al. (2022), provided evidence that SCG mediated CB hyperexcitability and sensitized the chemoreflex in spontaneously hypertensive (SH) rats. Electrical stimulation of the SCG in the working heart-brainstem preparation caused an enhanced chemoreflex response to NaCN infusions in the internal carotid artery (Felippe et al., 2022). *In vivo* bilateral SCGx in SH rats attenuated CB-evoked sympatho-hyperreflexia and the chemoreflex response to KCN infusion at both 5 days and 2 weeks post SCGx, suggesting that the SCG is involved in the CB chemoreflex sensitization in hypertensive animals (Felippe et al., 2022). However, involvement of the SCG in the sensitized chemoreflex during the recovery from ALI has never been explored. Our current data shows that SCGx attenuated the enhanced chemoreflex sensitivity in response to hypoxia and normoxic-hypercapnia challenge during the recovery from ALI. This study indicates that the SCG may be involved in the chemoreflex sensitzation in multiple pathological conditions including ALI. A potential limitation of our study is that we did not explore the downstream mechanisms underlying SCG-mediated chemoreflex sensitization during the recovery from ALI. It is well known that carotid body glomus cells can be sensitized by sympathetic neurotransmitters such as noepinephrine (NE) and ATP (Milsom and Sadig, 1983; Pijacka et al., 2016). During ALI, it is possible that hypoxia-evoked sympatho-excitation may cause more ATP and NE release from SCG to the carotid body, activating purinergic/adrenergic receptors expressed in glomus cells/carotid sinus nerve terminals and chronically facilitating chemoreceptor sensitization. This hypothesis needs to be tested in the future studies. In addition, ALI is an inflammatory disease, often associated with a systemic cytokine storm. Evidence of neuroinflammation in the stellate ganglion 4 weeks post-bleo has previously been reported (Hong et al., 2021). Such neuroinflammation may occur in the SCG as well. Whether the SCG-mediated neural inflammation sensitizes the CB as well as chemoreflex function during recovery from ALI needs to be investigated in the future studies. Another possibility is that ALI-induced systemic inflammation could cause neuroinflammation within the CB chemoreceptors that have recently been shown to sense systemic inflammatory molecules (Iturriaga et al., 2022). Evidence of ALI-induced neuroinflmmation within the CB will be investigated in the future.

It should be noted that although bilateral SCGx prevented the chemoreflex sensitization during the recovery from ALI, this intervention did not affect the chemoreflex function in normal rats. Neither did SCGx affect baseline ventilatory parameters (f_R_, V_t_ and V̇_E_) in normal rats. This was supported by a previous study by Getsy et al. (2021a) that reported the effects of bilateral SCGx on ventilatory parameters at rest and during hypoxic gas challenge in C57BL6 mice. Four days after bilateral SCGx, the ventilatory parameters at rest remained unchanged in both Sham-operated and SCGx mice, suggesting that the input from the SCG to CB and regions of upper airway and brainstem do not tonically regulate the baseline ventilatory parameters at rest. This finding was also supported by another study from the same group where they did not find any changes in resting baseline ventilation in C57BL6 mice with transection of the cervical sympathetic chain (Getsy et al., 2021b). Interestingly, these studies reported that during hypoxic challenge, SCGx mice demonstrated an attenuated ventilatory response in comparison to Sham-operated mice. Their later finding contradicts our finding that bilateral SCGx in non-ALI rats (healthy rats) caused no difference in chemoreflex activation evoked by hypoxia and normoxic-hypercapnia. This difference in response to hypoxia may be caused by multiple factors including the differences in animal species and the experimental time window post SCGx. Getsy et al. (2021b) measured the hypoxic ventilatory response at 4 days post SCGx whereas we assessed chemoreflex function at 2 weeks post SCGx. It is possible that within 2 weeks the animals may develop some compensatory mechanisms through which adaptation to the acute loss of SCG-mediated chemoreflex modulation occurs. This hypothesis needs to be confirmed in the future studies. Finally, although our evidence suggest the involvement of SCG in mediating chemoreflex sensitization during recovery from ALI, we noticed that SCGx did not alter resting ventilatory parameters (f_R_, V_t_ and V̇_E_) and apnea events in both acute ALI (W1) and during recovery (W4). During ALI, the mechanisms underlying increased respiratory drive are complicated, which may be due to direct stimulation of chemoreflexes, altered lung mechanics and inflammation (Spinelli et al., 2020). Occurrence of apnea and hypopnea are among many factors associated with increased sympathetic nerve activity, inflammation, and intermittent hypoxia, and may be caused by respiratory control instability (Javaheri et al., 2017). Given the fact that SCGx caused no change in resting ventilatory parameters as well as apneas during ALI, it is reasonable to speculate that the SCG may not be involved in tonic activation of the chemoreflex during ALI.

## Conclusion

This study provides evidence that bilateral SCGx largely abolished the chemoreflex sensitization during recovery from ALI without affecting chemoreflex function in the normal state. Further understanding of the underlying mechanism will provide important information for the long-term goal of developing novel targeted therapeutic approaches to pulmonary diseases to improve clinical outcomes.

## Data Availability

The raw data supporting the conclusion of this article will be made available by the authors, without undue reservation.
